# Complete Genome Sequence of a New Ruminococcaceae Bacterium Isolated from Anaerobic Biomass Hydrolysis

**DOI:** 10.1128/genomeA.00030-18

**Published:** 2018-04-05

**Authors:** Sarah Hahnke, Christian Abendroth, Thomas Langer, Francisco M. Codoñer, Patrice Ramm, Manuel Porcar, Olaf Luschnig, Michael Klocke

**Affiliations:** a Department of Bioengineering, Leibniz Institute for Agricultural Engineering and Bioeconomy (ATB), Potsdam, Germany; b Cavanilles Institute of Biodiversity and Evolutionary Biology, Universitat de Valencia, Paterna, Valencia, Spain; c Institute for Integrative Systems Biology (I^2^SysBio), Paterna, Valencia, Spain; d Robert Boyle Institut e.V., Jena, Germany; e Chair of Waste Management, Technische Universität Dresden, Pirna, Germany; f Lifesequencing SL, Parc Cientific Universitat de Valencia, Paterna, Valencia, Spain; g Darwin Bioprospecting Excellence, S.L. Parc Cientific Universitat de Valencia, Paterna, Valencia, Spain; h Bio H2 Energy GmbH, Jena, Germany

## Abstract

A new Ruminococcaceae bacterium, strain HV4-5-B5C, participating in the anaerobic digestion of grass, was isolated from a mesophilic two-stage laboratory-scale leach bed biogas system. The draft annotated genome sequence presented in this study and 16S rRNA gene sequence analysis indicated the affiliation of HV4-5-B5C with the family Ruminococcaceae outside recently described genera.

## GENOME ANNOUNCEMENT

Anaerobic digestion is a promising technology to generate biofuels and other products from biomass (1). However, depending on the lignocellulose content of the biomass, the degradation rate varies. Hence, a physical or biological biomass pretreatment can be helpful to support the microbial hydrolysis (2).

In order to enrich hydrolytic bacteria, a mesophilic (37°C) two-stage laboratory-scale leach bed system for biomethanation of freshly cut grass as the sole substrate was set up. From the process fluid, a new Ruminococcaceae bacterium, strain HV4-5-B5C, was isolated, presumably participating in the degradation of plant biomass. Isolation was performed under anoxic conditions on Anaerobic agar acc. to Brewer (Merck) after the diluted hydrolysate had been reincubated with microcrystalline cellulose as the sole carbon source.

Genome sequencing was performed using the Illumina NextSeq 500 platform. A Nextera XT library with a mean insert size of around 300 nucleotides (nt) was constructed and sequenced in a combination of 150-bp paired-end (PE) reads. In total, 21,629,863 PE sequences with a mean length of 150.32 nt were obtained. After quality filtering, 21.61 million PE sequences remained, with a mean Q value of 32.71. Genome assembly was conducted with software SPAdes version 3.10.1 (3) and default parameters using a *k*-mer of 127. A total of 5,019 contigs were obtained, including 76 contigs with a length of over 500 nt covering a total genome size of around 3.01 Mb, with an estimated GC content of 52.50%. The longest calculated contig was 581,597 nt, and the *N*_50_ value of the assembly was 360,256 nt.

The assembled sequences were annotated using the Prokka annotation pipeline version 1.11, including the prediction of tRNA, rRNA, and mRNA genes and signal peptides using the Aragorn, RNAmmer, Prodigal, and SignalP software (4–8).

The genome of strain HV4-5-B5C contained 2,945 elements, of which 2,878 were open reading frames (ORFs), with 2,325 canonical ORFs and 553 noncanonical ORFs, and 67 elements that encoded structural RNAs (sRNAs), with 6 for rRNA and 61 for tRNA.

Using BLAST, the determined contigs were compared with all genome sequences available at the NCBI database of complete bacterial genomes. Based on the percentage of conserved proteins (POCP) (9), the closest genome-sequenced relative of the novel Ruminococcaceae bacterium was Clostridium sporosphaeroides (38.31% identity), as also indicated by the average amino acid identity (AAI) (10) (54.72% identity). Analysis based on the average nucleotide sequence identity (ANI) (11) showed closest affiliation to Clostridium sporosphaeroides (68.83% identity), applying ANIBlast, and to Clostridium leptum (88.36% identity), applying ANI-MUMmer. Using the EzBioCloud identifier (12), 16S rRNA gene sequence comparisons revealed Caproiciproducens galactitolivorans BS-1^T^ to be the most closely affiliated strain, sharing 93.3% sequence identity. In summary, these results indicate that the novel bacterial strain represents a new species and possibly a new genus within the family Ruminococcaceae.

### Accession number(s).

Strain HV4-5-B5C was deposited at the German Collection of Microorganisms and Cell Cultures (DSMZ) under accession no. DSM-104463. The genome project was deposited at DDBJ/EMBL/GenBank under accession no. FXYJ02000001 to FXYJ02000076. The version described here is the first draft version.

## References

[1] Strong PJ, Kalyuzhnaya M, Silverman J, Clarke WP 2016 A methanotroph-based biorefinery: potential scenarios for generating multiple products from a single fermentation. Bioresour Technol 215:314–323. doi:10.1016/j.biortech.2016.04.099. 27146469

[2] Paudel SR, Banjara SP, Choi OK, Park KY, Kim YM, Lee JW 2017 Pretreatment of agricultural biomass for anaerobic digestion: current state and challenges. Bioresour Technol 245:1194–1205. doi:10.1016/j.biortech.2017.08.182. 28899674

[3] Bankevich A, Nurk S, Antipov D, Gurevich AA, Dvorkin M, Kulikov AS, Lesin VM, Nikolenko SI, Pham S, Prjibelski AD, Pyshkin AV, Sirotkin AV, Vyahhi N, Tesler G, Alekseyev MA, Pevzner PA 2012 SPAdes: a new genome assembly algorithm and its applications to single-cell sequencing. J Comput Biol 19:455–477. doi:10.1089/cmb.2012.0021. 22506599 PMC3342519

[4] Seemann T 2014 Prokka: rapid prokaryotic genome annotation. Bioinformatics 30:2068–2069. doi:10.1093/bioinformatics/btu153. 24642063

[5] Laslett D, Canback B 2004 ARAGORN, a program to detect tRNA genes and tmRNA genes in nucleotide sequences. Nucleic Acids Res 32:11–16. doi:10.1093/nar/gkh152. 14704338 PMC373265

[6] Lagesen K, Hallin P, Rødland EA, Stærfeldt H-H, Rognes T, Ussery DW 2007 RNAmmer: consistent and rapid annotation of ribosomal RNA genes. Nucleic Acids Res 35:3100–3108. doi:10.1093/nar/gkm160. 17452365 PMC1888812

[7] Hyatt D, Chen GL, Locascio PF, Land ML, Larimer FW, Hauser LJ 2010 Prodigal: prokaryotic gene recognition and translation initiation site identification. BMC Bioinformatics 11:119. doi:10.1186/1471-2105-11-119. 20211023 PMC2848648

[8] Petersen TN, Brunak S, von Heijne G, Nielsen H 2011 SignalP 4.0: discriminating signal peptides from transmembrane regions. Nat Methods 8:785–786. doi:10.1038/nmeth.1701. 21959131

[9] Qin QL, Xie BB, Zhang XY, Chen XL, Zhou BC, Zhou J, Oren A, Zhang YZ 2014 A proposed genus boundary for the prokaryotes based on genomic insights. J Bacteriol 196:2210–2215. doi:10.1128/JB.01688-14. 24706738 PMC4054180

[10] Konstantinidis KT, Tiedje JM 2005 Towards a genome-based taxonomy for prokaryotes. J Bacteriol 187:6258–6264. doi:10.1128/JB.187.18.6258-6264.2005. 16159757 PMC1236649

[11] Konstantinidis KT, Tiedje JM 2005 Genomic insights that advance the species definition for prokaryotes. Proc Natl Acad Sci U S A 102:2567–2572. doi:10.1073/pnas.0409727102. 15701695 PMC549018

[12] Kim OS, Cho YJ, Lee K, Yoon SH, Kim M, Na H, Park SC, Jeon YS, Lee JH, Yi H, Won S, Chun J 2012 Introducing EzTaxon-e: a prokaryotic 16S rRNA gene sequence database with phylotypes that represent uncultured species. Int J Syst Evol Microbiol 62:716–721. doi:10.1099/ijs.0.038075-0. 22140171

